# Evaluation of food insecurity and its association with food consumption and some variables among college students

**DOI:** 10.1186/s41043-023-00436-9

**Published:** 2023-09-01

**Authors:** Özge Mengi Celik, Caner Ozyildirim, Merve Seyda Karacil Ermumcu

**Affiliations:** 1 Department of Nutrition and Dietetics, Faculty of Health Sciences, University of Health Sciences, Ankara, Turkey; 2 Department of Nutrition and Dietetics, Faculty of Health Sciences, Akdeniz University, Antalya, Turkey

**Keywords:** Food insecurity, Food security, Food consumption frequency, Undergraduate students

## Abstract

**Background:**

Students are an important group threatened by food insecurity. Food insecurity among college students is one of the research topics that is attracting attention worldwide, and interest in this topic is growing by the day. Food insecurity has a negative impact on students' biological, mental and social health. This study aimed to determine the prevalence of food insecurity and correlate it with frequency of food consumption and some variables among undergraduate students.

**Methods:**

This descriptive-analytical study was conducted with 1149 university students at Akdeniz University, Antalya, Turkey. The demographic characteristics of the individuals (gender, age, living situation, income status, and health information), anthropometric measurements (body weight and height), nutritional habits, frequency of food consumption, and Household Food Insecurity Access Scale (HFIAS) were questioned. Statistical Package for the Social Sciences (SPSS) software was used for statistical analyses.

**Results:**

It was found that 13.1% of the students have mild, 13.0% moderate, and 9.4% severe food insecurity. The rate of individuals with and without food security differs according to gender, living situation, and income status (p < 0.05). There was a statistically significant correlations between the food insecurity score and age (p = 0.047), the number of main meals (p < 0.001) and snacks (0.007), and consumption frequency of certain foods (p < 0.05).

**Conclusions:**

The prevalance of students facing food insecurity was high. Individuals with food insecurity have a lower frequency of healthy food consumption than individuals with food security. Steps should be taken to ensure nutritional security among undergraduate students, who are the young adult population.

## Introduction

Food security is defined as meaning that all people, at all times, have physical, social, and economic access to sufficient, safe, and nutritious food that meets their food preferences and dietary needs for an active and healthy life. The concept of food security has four dimensions: food availability, access to food, utilization, and stability [1, 2]. Poverty, war, overpopulation, climate change, environmental problems, rising food prices, agricultural instability, and unequal social and economic policies are the main contributors to global food insecurity [2]. The number of malnourished people around the world and the number of starving individuals tend to increase day by day, and one of the main reasons for this is the increasing number of people who do not have access to food, that is, people with food insecurity. The increase in food insecurity since the last pandemic has made food insecurity a crisis compared to the pre-pandemic period [3]. The United Nations has set sustainable development goals to end all kinds of poverty as well as hunger and malnutrition by 2030, and thus it is aimed at preventing food insecurity [4, 5].

Some groups such as children, adolescents, the elderly and those with chronic diseases, have been reported to be at increased risk of food insecurity [1]. Undergraduate students are another important group at risk of food insecurity [6]. Food insecurity among college students worldwide, especially in the United States, is among the research topics that attract attention, and the interest in this subject has increased day by day [7]. Food insecurity among college students varies between 21 and 82% [8], and food insecurity is more common, especially in universities in rural areas [6, 9–12]. It is stated that younger and lower-income college students who do not receive government scholarships and do not have accommodation or dormitory facilities are at higher risk [13, 14]. Inadequate nutritional knowledge and food literacy cause an increase in the incidence of food insecurity among students. As can be seen, a high level of knowledge does not mean that food security is provided.

Students with food insecurity consume fruits, vegetables, fish, meat, dairy products, and legumes less frequently and in smaller amounts than students without food insecurity [8, 15]. It is stated that students with food insecurity have lower diet quality, poor mental health, and poor academic performance [1, 7, 16]. Therefore, students with food insecurity have a higher risk of weight gain and obesity [17]. Food insecurity negatively affects students in terms of mental and social health [18]. Long-term exposure of undergraduate students to nutritional insecurity (especially limited access to nutritious foods) may contribute to adverse health behaviors and, over time, the development of obesity, hypertension, diabetes, and cardiovascular disease-related comorbidities, and which may accelerate the incidence of chronic diseases. It has been suggested that food insecurity increases the rates of depression and anxiety, decreases the ability to concentrate [19], and causes low grade point averages in students [20, 21].

Studies on this subject are limited that reveals the relationship between food insecurity, and food preferences, and anthropometric measurements among undergraduate students in Turkiye, where the young population is in the majority. In this study, it was aimed to determine the prevalence of food insecurity, examine the socio-demographic characteristics associated with food insecurity, determine the food preferences of students according to food insecurity, and reveal its relationship with anthropometric measurements among undergraduate students.

## Methods

This is a cross-sectional analytical study. This study was conducted on 1149 students (599 females, 550 males) at Akdeniz University between May and July 2022. All students who agreed to participate in the study were included. At the end of the research, G*Power (version 3.1.9.7, Universitat Düsseldorf, Düsseldorf, Germany) program was used for post-hoc power analysis. The power of the study (1−β) was found to be 97% (Alpha 5% and confidence interval 95%). Before starting the study, ethics approval with the decision number KAEK-350 dated May 11, 2022, was obtained from the Clinical Research Ethics Committee of Akdeniz University Faculty of Medicine. All procedures in the study were carried out according to the Declaration of Helsinki. The research data were collected face-to-face with the questionnaire prepared by the researchers.

### Socio-demographic and health status

We questioned gender, living situation and income. We would like to point out that instead of determining the exact income levels to question the income status, we asked the students to indicate their income-expenditure status (because we think this question may be sensitive for students). For this purpose, we used the following questions: (1) My income is higher than my expenses, (2) my income is equal to my expenses, and (3) my income is less than my expenses.

Chronic disease status was questioned as whether there is a chronic disease (yes/no); if yes, which disease you were diagnosed with.

### Nutritional habits and frequency of food consumption

The food consumption of individuals in the last month was evaluated using the frequency form created by the researchers. Food consumption frequency questionnaire was not adapted from a standart questionnaire. While recording the food frequency, a photographic food atlas containing detailed food quantities and food groups was used [22]. Food consumption frequency included the categories of milk and dairy products, meat and meat products, cereals and legumes, fruit and vegetables, fats and oils, sugar and sweets, beverages and packaged products. The food consumption frequency questionnaire is presented in the supplementary table. Consumption three times a week or more was classified as 'very frequent', consumption 1–2 times a week as 'frequent', once in 15 days and once a month as 'rarely', and no consumption as 'never'. In addition to the number of main and snack meals, the meals eaten out and their frequency were also questioned.

### Anthropometric measurements

The body weight (kg) and height (cm) of the individuals were evaluated by the researchers using standard measurement protocols. Body mass measurements (kg) were taken using a calibrated and 0.5 kg sensitive scale device. Height was measured in the standing position without shoes using a stadiometer with an accuracy of 0.1 cm [23]. Body mass index (BMI) was calculated by dividing body weight by the square of height. Individuals with a body mass index below 18.50 kg/m^2^ were classified as underweight, between 18.50 and 24.99 kg/m^2^ as normal, between 25.0 and 29.99 kg/m^2^ as overweight, and over 30.0 kg/m^2^ as obese [24].

### Food insecurity

The level of food insecurity of individuals was evaluated with the 'Household Food Insecurity Access Scale (HFIAS)’ [25]. The Turkish validity and reliability study of the scale was conducted in 2018 [26]. The Cronbach's alpha value of the Turkish version of this scale was 0.876. There are nine questions on the scale that evaluate the situation of food insecurity in the last month. The maximum score that can be obtained from the scale is 27, and the minimum score is 0. The HFIAS score is a measure of household food insecurity over the past 30 days. Higher scores indicate higher severity of household food insecurity. Individuals' food insecurity status was classified into 4 groups according to the total score: food security, mild food insecurity, moderate food insecurity, and severe food insecurity. Food insecurity was categorised as described in the scale [25].

### Statistics

SPSS 22.0 statistical package program was used to evaluate the data obtained from the study. Distribution analysis of the data was performed using the histogram, coefficient of variation ratio, Skewness, Kurtosis, and Shapiro–Wilk tests. Relationships between numerical variables are given by the Spearman correlation coefficient. Mann Whitney U test was used to compare two independent groups that were not normally distributed. Spearmen correlation was used for the correlation of non-normally distributed data. Chi-square analysis was used to compare the qualitative data and determine the differences between the groups. Regression analysis was performed for food insecurity prediction. For the linear regression analysis, the non-normally distributed variables were transformed using logarithmic transformation to more closely approximate normal distribution. The results were evaluated at the 95% confidence interval, statistically at a p < 0.05 significance level.

## Results

The study was completed with 1149 undergraduate students (599 females and 550 males). The mean age of the individuals was 21.5 ± 2.31 years. Half of the students (50.7%) reported living in a dormitory and 23.8% reported that their income was less than their expenses. The majority of individuals (72.6%) were in normal weight according to BMI classification. 22.7% of the students had a chronic disease (Table 1).

**Table 1:** Descriptive characteristics of participants

Variables	Number (%)
Gender	
Female	599 (52.1%)
Male	550 (47.9%)
Living situation	
Home	567 (49.3%)
Dormitory	582 (50.7%)
Income status	
Income more than expenses	229 (19.9%)
Income equal to expenses	646 (56.3%)
Income less than expenses	274 (23.8%)
Chronic disease status	
Yes	261 (22.7%)
No	888 (77.3%)
	$\overline {{\text{X}}}$ ± SD
BMI classification	
Underweight(< 18.50 kg/m^2^)	105 (9.1%)
Normal (18.50–24.99 kg/m^2^)	834 (72.6%)
Overweight (25.00–29.99 kg/m^2^)	171 (14.9%)
Obese (≥ 30.0 kg/m^2^)	39 (3.4%)

$\bar{X}$ mean; *SD* Standard deviation; *BMI* Body mass index

The average number of main meals for the students was 2.4 ± 0.60, and the number of snacks was 1.6 ± 0.97. While 36.1% of the students stated that they did not consume their morning meal outside, the majority of the students often and very often consume lunch (73.0%) and dinner meals (57.1%) outside. 13.1% of the students had mild, 13.0% moderate, and 9.4% severe food insecurity. In general, 35.5% of the students had food insecurity (Table 2).

**Table 2:** Nutritional habits and food security status of participants

	X̅ ± SD
Number of main meals	2.4 ± 0.60
Number of snacks	1.6 ± 0.97
HFIAS score	1.94 ± 3.60
	Number (%)
*Frequency of eating out*	
Breakfast	
Very often	226 (19.7%)
Often	199 (17.3%)
Rarely	309 (26.9%)
Never	415 (36.1%)
Lunch	
Very often	668 (58.1%)
Often	171 (14.9%)
Rarely	173 (15.1%)
Never	137 (11.9%)
Dinner	
Very often	367 (31.9%)
Often	290 (25.3%)
Rarely	316 (27.5%)
Never	176 (15.3%)
*Evaluation of the food insecurity*	
Food security	741 (64.5%)
Food insecurity	408 (35.5%)
Mild food insecurity	151 (13.1%)
Moderate food insecurity	149 (13.0%)
Severe food insecurity	108 (9.4%)

$\bar{X}$ mean; *SD* Standard deviation; *HFIAS* Household Food Insecurity Access Scale

A statistically significant difference was found in terms of the rate of individuals with and without food security according to gender, living situation, and income status (p < 0.05) (Table 3). There was no statistically significant difference in the frequency of meals consumed outside according to food security status (data not shown in the table).

**Table 3:** Relationship of food insecurity with sociodemographic and anthropometric variables

Variables	Food insecurity status		p-Value
	Food security	Food insecurity	
Gender	741 (64.5%)	408 (35.5%)	
Female	408 (68.1%)	191 (31.9%)	
Male	333 (60.5%)	217 (39.5%)	
Living situation			0.007*
Home	405 (71.4%)	162 (28.6%)	
Dormitory	336 (57.7%)	246 (42.3%)	
Income status			< 0.001*
Income more than expenses	180 (78.6%)	49 (21.4%)	
Income equal to expenses	440 (68.1%)	206 (31.9%)	
Income less than expenses	121 (44.2%)	153 (55.8%)	
Chronic disease status			< 0.001*
Yes	161 (61.7%)	100 (38.3%)	
No	580 (65.3%)	308 (34.7%)	
Body weight (kg)			0.281
Female	58.3 ± 0.57	57.5 ± 0.76	0.256
Male	76.4 ± 0.62	76.2 ± 0.77	0.167
BMI (kg/m^2^)	22.5 ± 0.13	22.5 ± 0.16	0.299
BMI classification (number, %)			0.234
Underweight (< 18.5 kg/m^2^)	62 (8.4%)	43 (10.5%)	
Normal (18.5–24.99 kg/m^2^)	550 (74.2%)	284 (69.6%)	
Overweight (≥ 25.00–29.99 kg/m^2^)	102 (13.8%)	69 (16.9%)	
Obese (≥ 30.0 kg/m^2^)	27 (3.6%)	12 (2.9%)	

*BMI* Body mass index

*Significant at p-value < 0.05

A statistically significant difference was found between individuals with and without food security in terms of the frequency of consumption of milk, yogurt, cheese, red meat, fish, oil seeds, fresh fruits, dried fruits, green leafy vegetables, dried vegetables, olive oil, sunflower oil, and other liquid oils. (p < 0.05) (Table 4).

**Table 4:** Assessment of food consumption frequency according to food insecurity status

Foods	Food insecurity status	Very often	Often	Rarely	Never	p-Value
Milk	Food security Food insecurity	306 (41.3%) 107 (26.2%)	189 (25.5%) 129 (31.6%)	168 (22.7%) 114 (27.9%)	78 (10.5%) 58 (14.2%)	< 0.001*
Yogurt	Food security Food insecurity	445 (60.1%) 165 (40.4%)	192 (25.9%) 156 (38.2%)	88 (11.9%) 73 (17.9%)	16 (2.2%) 14 (3.4%)	0.001*
Cheese	Food security Food insecurity	537 (72.5%) 272 (66.7%)	114 (15.4%) 67 (16.4%)	54 (7.3%) 51 (12.5%)	36 (4.9%) 18 (4.4%)	0.025*
Kefir	Food security Food insecurity	61 (8.2%) 28 (6.9%)	69 (9.3%) 26 (6.4%)	213 (28.7%) 102 (25.0%)	398 (53.7%) 252 (61.8%)	0.051
Red meat	Food security Food insecurity	247 (33.3%) 78 (19.1%)	253 (34.1%) 144 (35.3%)	184 (24.8%) 135 (33.1%)	57 (7.7%) 51 (12.5%)	0.001*
Chicken	Food security Food insecurity	364 (49.1%) 198 (48.5%)	257 (34.7%) 127 (31.1%)	105 (14.2%) 71 (17.4%)	15 (2.0%) 12 (2.9%)	0.290
Fish	Food security Food insecurity	54 (7.3%) 16 (3.9%)	94 (12.7%) 26 (6.4%)	377 (50.9%) 217 (53.2%)	216 (29.1%) 149 (36.5%)	0.001*
Processed meat products	Food security Food insecurity	217 (29.3%) 138 (33.8%)	163 (22.0%) 93 (22.8%)	221 (29.8%) 108 (26.5%)	140 (18.9%) 69 (16.9%)	0.330
Egg	Food security Food insecurity	517 (69.8%) 283 (69.4%)	105 (14.2%) 53 (13.0%)	74 (10.0%) 49 (12.0%)	45 (6.1%) 23 (5.6%)	0.714
Legumes	Food security Food insecurity	309 (41.7%) 138 (33.8%)	288 (38.9%) 172 (42.2%)	127 (17.1%) 85 (20.8%)	17 (2.3%) 13 (3.2%)	0.054
Oil seeds	Food security Food insecurity	292 (39.4%) 104 (25.5%)	223 (30.1%) 119 (29.2%)	178 (24.0%) 146 (35.8%)	48 (6.5%) 39 (9.6%)	< 0.001*
Bread	Food security Food insecurity	613 (82.7%) 345 (84.6%)	61 (8.2%) 28 (6.9%)	43 (5.8%) 25 (6.1%)	24 (3.2%) 10 (2.5%)	0.719
Pasta, noodle	Food security Food insecurity	400 (54.0%) 212 (52.0%)	251 (33.9%) 142 (34.8%)	78 (10.5%) 45 (11.0%)	12 (1.6%) 9 (2.2%)	0.843
Rice	Food security Food insecurity	371 (50.1%) 210 (51.5%)	275 (37.1%) 147 (36.0%)	81 (10.9%) 40 (9.8%)	14 (1.9%) 11 (2.7%)	0.731
Bulgur wheat	Food security Food insecurity	283 (38.2%) 140 (34.3%)	297 (40.1%) 151 (37.0%)	127 (17.1%) 94 (23.0%)	34 (4.6%) 23 (5.6%)	0.070
Fresh fruit	Food security Food insecurity	431 (58.2%) 173 (42.4%)	183 (24.7%) 122 (29.9%)	99 (13.4%) 99 (24.3%)	28 (3.8%) 14 (3.4%)	< 0.001*
Dried fruit	Food security Food insecurity	154 (20.8%) 51 (12.5%)	131 (17.7%) 54 (13.2%)	257 (34.7%) 161 (39.5%)	199 (26.9%) 142 (34.8%)	< 0.001*
Green leafy vegetables	Food security Food insecurity	305 (41.2%) 130 (31.9%)	212 (28.6%) 117 (28.7%)	170 (22.9%) 123 (30.1%)	54 (7.3%) 38 (9.3%)	0.005*
Other fresh vegetables	Food security Food insecurity	295 (39.8%) 157 (38.5%)	231 (31.2%) 131 (32.1%)	150 (20.2%) 95 (23.3%)	65 (8.8%) 25 (6.1%)	0.298
Dried vegetables	Food security Food insecurity	87 (11.7%) 25 (6.1%)	89 (12.0%) 35 (8.6%)	228 (30.8%) 129 (31.6%)	337 (45.5%) 219 (53.7%)	0.002*
Olive oil	Food security Food insecurity	388 (52.4%) 175 (42.9%)	116 (15.7%) 57 (14.0%)	132 (17.8%) 105 (25.7%)	105 (14.2%) 71 (17.4%)	0.002*
Sunflower oil	Food security Food insecurity	469 (63.3%) 295 (72.3%)	110 (14.8%) 51 (12.5%)	94 (12.7%) 35 (8.6%)	68 (9.2%) 27 (6.6%)	0.016*
Other vegetable oils	Food security Food insecurity	100 (13.5%) 33 (8.1%)	55 (7.4%) 12 (2.9%)	123 (16.6%) 72 (17.6%)	463 (62.5%) 291 (71.3%)	< 0.001*
Fast-foods	Food security Food insecurity	308 (41.6%) 163 (40.0%)	230 (31.0%) 109 (26.7%)	182 (24.6%) 123 (30.1%)	21 (2.8%) 13 (3.2%)	0.169
Packaged foods	Food security Food insecurity	400 (54.0%) 221 (54.2%)	165 (22.3%) 90 (22.1%)	142 (19.2%) 83 (20.3%)	34 (4.6%) 14 (3.4%)	0.792
Frozen foods	Food security Food insecurity	110 (14.8%) 64 (15.7%)	103 (13.9%) 66 (16.2%)	277 (37.4%) 148 (36.3%)	251 (33.9%) 130 (31.9%)	0.692
Canned foods	Food security Food insecurity	72 (9.7%) 39 (9.6%)	84 (11.3%) 53 (13.0%)	246 (33.2%) 143 (35.0%)	339 (45.7%) 173 (42.4%)	0.673

*Significant at p-value < 0.05

Consumption frequency of certain foods (milk, yogurt, cheese, kefir, red meat, fish, legumes, oilseeds, bulgur, fresh fruits, dried fruits, green leafy vegetables, other fresh vegetables, dried vegetables, olive oil and other oils), age, and the number of meals and snacks were negatively correlated with the HFIAS score (p < 0.05). A statistically significant positive correlation was found between the HFIAS score and the consumption frequency of sunflower oil (p < 0.05) (Table 5).

**Table 5:** Evaluation of the relationship between food insecurity and studied variables

	Household food insecurity accession scale score
Age (years)	r = − 0.059 p = 0.047*
BMI (kg/m^2^)	r = 0.042 p = 0.152
Number of main meals	r = − 0.104 p < 0.001*
Number of snacks	r = − 0.082 p = 0.007*
Frequency of eating out	
Breakfast	r = − 0.033 p = 0.269
Lunch	r = − 0.040 p = 0.178
Dinner	r = − 0.007 p = 0.814

*BMI* Body mass index

*Significant at P-value < 0.05

When the factors that could affect the Household Food Insecurity Access Scale total score was evaluated with linear regression analysis, the model was deemed important (R^2^ = 0.216; p < 0.001). It was determined that age, gender and income status affected the food insecurity (p < 0.05) (Table 6).

**Table 6:** Linear regression analysis for food insecurity prediction

Model	Household food insecurity access scale (HFIAS) total score		
	Beta	t	p-Value
Age (years)	− 0.060	− 2.106	0.003*
Gender	0.079	2.753	< 0.001*
Income status	− 0.256	− 8.999	< 0.001*
		**R**^**2**^ = 0.216; p < 0.001*	

Variables values: Gender (Male = 1, Female = 0), Income Status (Income less than expenses = 1, Income equal to expenses = 2, Income more than expenses = 3)

*Significant at p-value < 0.05

## Discussion

The aim of this study was to determine the prevalence of food insecurity, examine the socio-demographic characteristics associated with food insecurity, determine students' food preferences according to food insecurity, and determine its relationship with anthropometric measurements in college students. To our knowledge, this is the first study on food insecurity and related factors among Turkish college students.

We found that age, gender and income status had an effect on scores on the Household Food Insecurity Access Scale. It has been reported that the problem of food insecurity is increasing among students in middle- and high-income countries [6, 9, 15]. According to the results of the Nutrition and Health Survey conducted at the national level in Turkiye, the frequency of people worrying about not having enough food due to a lack of money and other resources in the last year was 23.4%, and the frequency of people experiencing a decrease in the type of food consumed was 22.8% [27]. We found food insecurity in 35.5% of students (13.1% mild, 13.0% moderate, and 9.4% severe), which is in line with the frequencies reported in other studies [26, 28, 29]. The prevalence of food insecurity among college students was reported as 30.8% in Mexico (16.3% for mild-FI, 8.8% for moderate-FI, and 5.7% for severe-FI) [28], 31% in the United States of America [29], and 43.6% in Turkiye (14.5% for mild-FI, 14.8% for moderate-FI, and 14.3% for severe-FI) [26]. In general, food insecurity among college students is estimated to range between 21 and 82% [8]. In other words, Turkish college students are at considerable risk in terms of food security. In addition, we found that male students were more likely to be food insecure than female students. However, studies show that food insecurity can be high for both sexes [30, 31].

Many studies have reported that consumption of fruit, vegetables, and whole grains decreases and consumption of sugar-sweetened beverages, saturated fat, and salt increases among food insecure students [8, 15, 31, 32]. There is also an increased risk of micronutrient deficiencies among people who are food insecure [33]. Consistent with the literature, we found that the frequency of consumption of milk, yogurt, cheese, red meat, fish, oilseeds, fresh fruit, dried fruit, green leafy vegetables, dried vegetables, and olive oil was lower among food-insecure students. The fact that food insecurity is associated with poorer diet quality may be a factor in this [34, 35]. Because food-insecure students often had to choose cheaper and less healthy alternatives [36]. A very low level of food security was associated with a 10% lower intake of eicosapentaenoic acid and docosahexaenoic acid [35]. All of this may have contributed to the high consumption of sunflower oil by students who are food insecure. The additional barriers faced by food insecure students may be one of the reasons for these differences in food consumption. Some of these barriers included a lack of visibility of food insecurity on the campuses of the participants and in their communities [18]. Food insecurity and the use of resources to address food insecurity were perceived by participants as stigmatising. This had an impact on the effectiveness of campus and community efforts [18]. Young people with very low food security were significantly more likely than those with high food security to report that healthy food is expensive, that there is a lack of healthy food available at home, and that no one prepares healthy food at home [37]. For these reasons, self-efficacy in food preparation and cooking is low among food insecure students [32, 38]. Our results show that the majority of students frequently eat out for lunch and dinner. Leung et al. [32] reported that in the context of their family, work, school, and social lives, food insecure students do not have time to plan or prepare their meals. In one report, the increase in the number of hours worked per week by college students was also associated with an increase in their food insecurity [39]. Therefore, we believe that one of the reasons that drives food-insecure students towards unhealthy foods is their income. College students stated that unhealthy foods, especially fast food, candy, and chocolate, are more affordable [40, 41]. Ukegbu et al. [4] reported that income status is related to food insecurity among college students. Consistently, we found that those whose income was higher than their expenditure were less likely to be food insecure. Students with an income equal to or less than their expenses are often able to choose less healthy foods, sacrificing food quality to save money [8]. These students prioritize cheapness by eating smaller portions or buying less nutritious food [42]. They use these methods as coping mechanisms [42]. In addition, these negative effects of food insecurity cause students to feel stressed about not having enough to eat and to avoid socializing, which has a negative impact on their mental health [43]. Food insecurity creates feelings of guilt, anxiety, and loneliness, which affect both students’ academic and social lives [42]. As high stress is also associated with increased consumption of ultra-processed foods [44], poor mental health may increase unhealthy food preferences in students with food insecurity.

In fact, studies show that food insecurity is a significant risk factor for obesity [45, 46]. Food-insecure students are 2–3 times more likely to be obese [47]. However, our results and some others [7, 48, 49] showed that food insecurity was not associated with BMI. The fact that our sample represents only one campus may explain this difference. The prevalence of food insecurity may vary from one region to another. Furthermore, while 72.6% of students in our population were in the normal BMI range, only 3.4% were classified as obese. Mohamadpour et al. [49] found an association between waist circumference and food insecurity, although there was no association between BMI groups and food insecurity. Therefore, the use of different measures may also be related to this finding. Moreover, we studied a young population, and we do not know how long students have been exposed to food insecurity. This may also be important because exposure to food insecurity at a young age is associated with greater BMI increases later in life [50]. Finally, the fact that food insecurity is unrelated to BMI in this study does not mean that food insecurity is not associated with health risks. It is stated that although students with food insecurity consume foods that are inadequate in terms of diet quality, they exceed their daily energy needs with the foods they prefer [47]. This may increase the risk of weight gain and obesity by causing high energy intake and may lead to the development of non-communicable diseases [4]. It has been shown that food insecurity may be associated with deterioration in mental health [49], impaired eating behaviors [7], and the risk of cardiovascular disease [51]. Physical, mental, emotional and social health is negatively affected in college students with food insecurity [18].

This study had several limitations. The cross-sectional design of this study only allowed for the examination of associations rather than potential causality between food insecurity, diet, health or other correlates. The fact that our food consumption frequency questionnaire was not validated is one of the important limitations of the study. Respondents were able to choose whether or not to participate in the survey, which may have introduced selection bias. In addition, future studies should include other variables that may be associated with food security, such as cooking skills, employment status, mental health and physical activity.

## Conclusions

Food insecurity is a growing global public health problem and a major barrier to adequate and balanced nutrition. Food insecurity is also a significant problem among college students. In this study, food insecurity was shown to reduce the consumption of key food groups among college students. The number of meals consumed also decreased in parallel with food insecurity. We also found that food insecurity was not associated with BMI. Therefore, food insecurity may be a risk for everyone, regardless of their weight classification. Eliminating food insecurity among college students can prevent inadequate and unbalanced diets.

## Data Availability

Not applicable.
